# The type of exercise most beneficial for quality of life in people with atrial fibrillation: a network meta-analysis

**DOI:** 10.3389/fcvm.2024.1509304

**Published:** 2025-01-09

**Authors:** Zhen Yang, Xiaoting Qi, Gaopeng Li, Na Wu, Bingwen Qi, Mengyang Yuan, Yaxin Wang, Guangling Hu, Qiaofang Yang

**Affiliations:** ^1^ Henan Provincial People’s Hospital, Fuwai Central China Cardiovascular Hospital, Zhengzhou Key Laboratory of Cardiovascular Nursing, Zhengzhou, Henan, China; ^2^ Department of Cardiovascular Physiology Faculty of Medicine, Kagawa University, Kagawa, Japan

**Keywords:** network meta-analysis, exercise, atrial fibrillation, quality of life, rehabilitation

## Abstract

**Introduction:**

Atrial fibrillation (AF) significantly detracts from health-related quality of life (HRQoL). Despite the promotion of exercise interventions for managing AF, the effectiveness of different exercise modalities remains to be clearly defined. This systematic review and network meta-analysis aims to evaluate the comparative effectiveness of various modes of exercise interventions on HRQoL in AF patients.

**Methods:**

A random-effect network meta-analysis was performed. We conducted comprehensive searches across multiple databases, including PubMed, Web of Science, Embase, Cochrane Library, Scopus, and Chinese databases such as CNKI, WanFang Data, and VIP. The review included only randomized controlled trials (RCTs) that investigated the effects of exercise interventions on HRQoL among individuals diagnosed with AF.

**Results:**

The network meta-analysis (NMA) incorporated 12 studies, of which five presented some concerns regarding risk of bias and one exhibited a high risk of bias. For total HRQoL in AF patients, aerobic exercise, and cardiac rehabilitation (CR) yielded standardised mean differences of 0.60 (95% CI: 0.02–1.13) and 0.59 (95% CI: 0.20–0.99), respectively. For the physical component of HRQoL, CR was most efficacious, demonstrating the highest Surface Under the Cumulative RAnking curve (SUCRA) value of 77%. For the mental component of HRQoL, high-intensity interval training (HIIT) was superior, with the highest SUCRA value of 90.7%.

**Conclusions:**

Both aerobic exercise and CR effectively improve the physical and mental dimensions of HRQoL as well as overall HRQoL in patients with AF. However, for the mental component of HRQoL, HIIT was identified as the most effective intervention.

## Introduction

At present, the prevalent therapeutic strategies for atrial fibrillation (AF) encompass pharmacological management and radiofrequency ablation. AF ablation, as a fundamental intervention, has been shown to significantly improve the quality of life (QoL) for AF patients by reducing symptoms and arrhythmia burden over the long term (1). Nonetheless, research indicates that the long-term therapeutic yield of these modalities, including pharmacological treatments, remains relatively inconclusive for sustained improvement in some cases (2). Recently, a proliferation of guidelines (3–5) has advocated for exercise as a remedial measure for patients diagnosed with AF. Empirical studies have corroborated that judicious exercise can attenuate the frequency of AF (6–9). Relative to AF patients who are sedentary, even transient periods of exercise can ameliorate the disease burden and enhance their health-related quality of life (HRQoL) (10, 11).

The impact of exercise on patients with AF is primarily attributable to two principal mechanisms. Firstly, exercise training can ameliorate atrial remodeling. Atrial enlargement is considered a hazard factor for novel AF episodes. Literature suggests that augmentation in atrial volume could precipitate atrial remodeling and escalate the progression of AF (12). Research has demonstrated that the mean left atrial volume in patients who experience AF recurrence post-radiofrequency ablation surpasses that of those who do not exhibit a recurrence (12). Exercise training, by mitigating intra-atrial pressure and volume, can reverse left atrial remodeling, reduce the instability of the sinoatrial node and proximal structures, thereby diminishing the prevalence of AF (13). Secondly, exercise training can facilitate autonomic equilibrium. The onset of AF may be a consequence of hyperactive autonomic reflexes and a compound effect of AF risk determinants such as obesity and hypertension, thus augmenting the propensity for arrhythmia (14). Hence, modulation of the autonomic nervous system may aid in reducing AF episodes. Studies have revealed that exercise training can decrease the resting heart rate and bolster cardiac vagal tone, thereby achieving the objective of autonomic regulation (15).

While empirical evidence substantiates the beneficial impact of exercise on the HRQoL in individuals diagnosed with AF, the question regarding the superior exercise modality for maximizing these benefits remains uncharted territory. Consequently, we have executed a NMA to meticulously investigate the differential implications of assorted types of exercise on the HRQoL in atrial fibrillation patients.

## Methods

This systematic review, along with the NMA, is following strictly to the guidelines stipulated by the Preferred Reporting Items for Systematic Reviews and Meta-Analyses for Network Meta-Analyses (PRISMA-NMA) (16), in conjunction with the principles outlined in the Cochrane Collaboration Handbook for Systematic Reviews (17). Additionally, the procedural blueprint for this research is registered with Prospero (CRD42023444482).

### Search strategy and selection criteria

**Figure 1:**
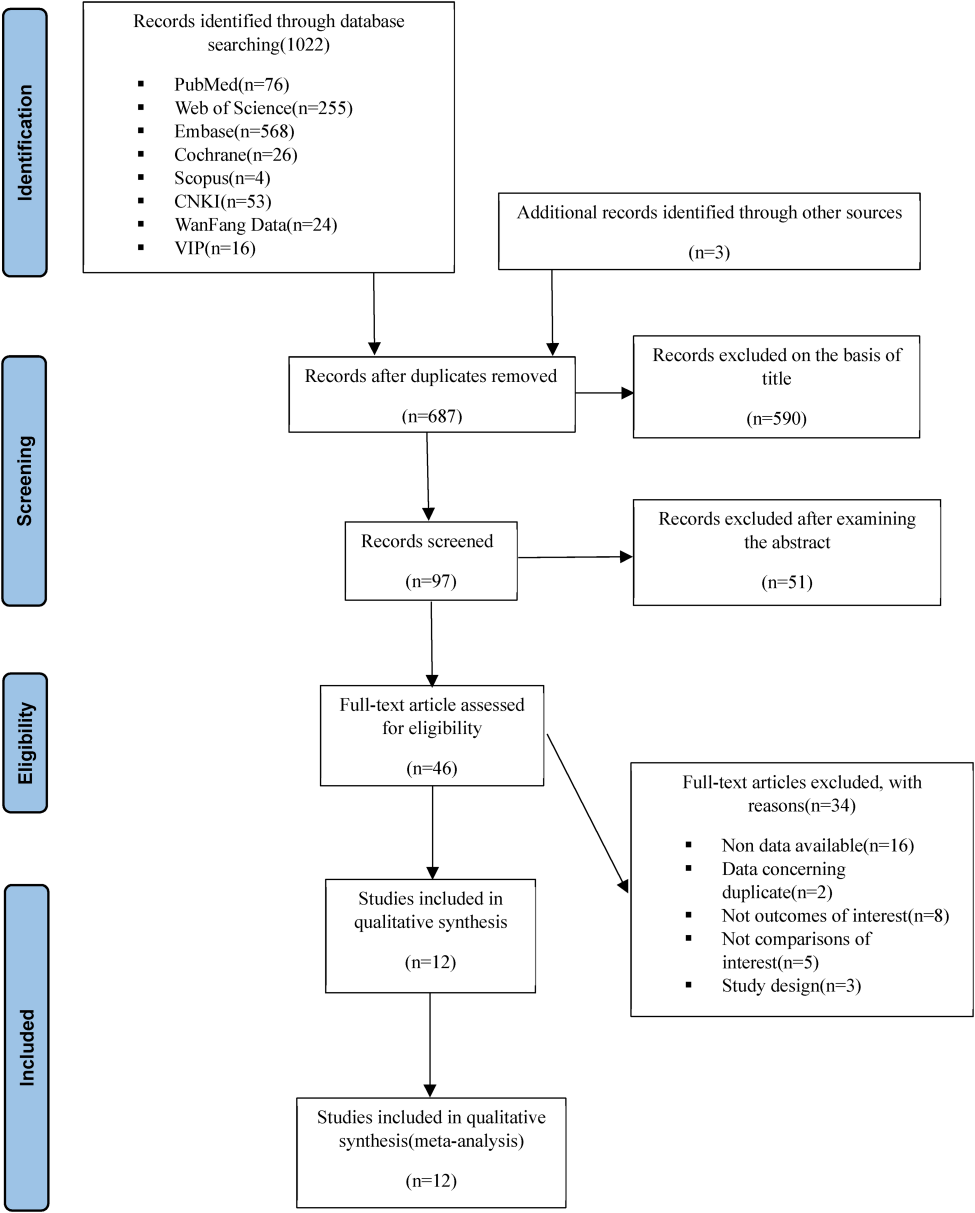
Flow of the selection of articles.

Two independent reviewers (YW and MY) embarked on a comprehensive literature (the screening of the literature is shown in Figure 1) search across several databases that span both English (PubMed, Web of Science, Embase, the Cochrane Library, Scopus) and Chinese languages (CNKI, WanFang Data, VIP), concluding their search on July 1, 2023. In instances where disparities emerged, a third-party arbitration (XQ) was sought to reach consensus. The search methodology was thoughtfully devised to encapsulate pertinent keywords related to: (1) Atrial Fibrillation, (2) Exercise, (3) Quality of Life, and (4) Randomized Controlled Trials. Further depth was added to our review by meticulously sifting through the reference lists of articles assimilated into this NMA, as well as prior reviews, with a view to discern any additional studies of relevance that may have been overlooked in the initial search.

### Eligibility

The present study undertakes an in-depth exploration of the implications of physical exercise on the HRQoL in patients diagnosed with AF. The selection criteria for this investigation were stringently defined to ensure robustness and specificity of the results. These criteria encompassed: (1) Inclusion of patients diagnosed with atrial fibrillation; (2) Restriction to RCTs to ensure high levels of evidence; (3) Examination of physical exercise interventions, unrestricted by intensity, duration, or frequency, offering broad implications for varying exercise regimes; (4) Comparative analysis of exercise interventions against other categories or control groups receiving standard care; (5) Primacy given to the evaluation of HRQoL as the primary outcome measure.

Studies were excluded under the following conditions: (1) Where physical exercise was amalgamated with other multidisciplinary interventions, such as pharmacological treatments or nutritional modifications, as these could potentially dilute the impact of exercise alone; (2) Where the interventions were purely educational in nature, as the study seeks to gauge the impact of physical activity; (3) Where the type or nature of exercise was indeterminate, to ensure the reproducibility and specificity of findings; (4) Where there was inadequate reporting of data, precluding reliable calculation of effect size; (5) Where the source of data was a conference abstract lacking full-text publication, to maintain the comprehensive and robust nature of the data; (6) Where publications were not scribed in either English or Chinese, to ensure consistency and reliability of data interpretation.

### Data extraction

Employing a prespecified table, both reviewers (ZY and XQ) undertook an independent extraction of the data, with any ensuing disputes deftly resolved by a senior researcher, thereby ensuring an unbiased and rigorous process. The extracted data encompassed both demographic and outcome parameters, inclusive of the year of publication, country of origin, study design, sample size, age range of the participants, nature of the interventions and controls, as well as the outcome measures (quality of life, physical components, mental components, among others).

### Classification of the intervention

In this NMA, the exercise intervention modalities were meticulously classified as follows:

• Aerobic Exercise: Interventions designed to elevate heart rate and energy expenditure through sustained, rhythmic activities, such as cycling, running, and brisk walking.
• Resistance Training: Aimed at improving muscular strength and endurance by engaging specific muscle groups against resistance, such as weights, resistance bands, or body weight.
• High-Intensity Interval Training (HIIT): A regimen involving alternating bouts of high-intensity aerobic exercise (e.g., sprinting, high-intensity cycling) and low-intensity recovery periods.
• Cardiac Rehabilitation (CR) Exercise: A structured program based on exercise and lifestyle modifications specifically tailored for cardiac patients, including those with AF. CR combines aerobic, resistance, and flexibility exercises with patient education and counseling to reduce cardiovascular risk.
• Yoga (Yoga-Based Exercise Training): Exercise based on physical postures, controlled breathing, and mindfulness practices.
• Usual Care: Involves maintaining routine basic treatment without a specific exercise regimen, allowing patients to follow usual lifestyle habits without additional structured exercise interventions.

### Outcome

In all included studies, the HRQoL outcomes were gauged through one or more self-reported questionnaires, with a majority indicating that higher scores denote superior HRQoL. However, in the event of a study utilising an inverse scoring system (higher scores representing poorer HRQoL), the mean value of each group was multiplied by −1 to align with the conventional interpretation. When a composite HRQoL score was not provided by the scale in use (for instance, in the SF-36 questionnaire), we consolidated the individual dimensions into a single composite measure, concurrently calculating its standard mean deviation. Ultimately, the analyses incorporated overall quality of life, physical components, and mental components, thereby providing a comprehensive and multidimensional evaluation of HRQoL.

### Equity, diversity and inclusion statement

Our research team is steadfastly dedicated to advancing diversity, equity, and inclusion within the scope of our clinical endeavors, research initiatives, and educational programs. We recognize that systemic disparities and inherent biases have the potential to influence the integrity of the research process. In response to these challenges, we have initiated measures to mitigate these concerns. Accordingly, the procedures of data extraction, processing, and interpretation have been conducted with minimal constraints to ensure that the outcomes are a comprehensive and authentic representation of reality, embracing the broadest possible spectrum of diversity to enhance the accuracy and inclusivity of our findings.

### Risk of bias assessment

Two researchers (MY and YW) independently appraised the risk of bias inherent in the incorporated RCTs, employing the Cochrane Collaboration's Risk of Bias 2 tool (RoB-2) as a gold-standard measure (18). Any divergences in assessment were addressed through consensus-building with a third reviewer (XQ), ensuring an unbiased and rigorous appraisal process. The RoB-2 tool methodically dissects the risk of bias across five distinct domains: (1) Bias emerging from the randomization process; (2) Bias attributed to deviations from intended interventions; (3) Bias originating from missing outcome data; (4) Bias in the measurement of outcomes; and (5) Bias in the selection of the reported result. The cumulative risk of bias was categorized as “low” if every domain of the study was scored as such. If at least one domain raised “some concerns”, the overall bias was labelled correspondingly. The study was classified as having a “high risk of bias” if any single domain was rated “high risk” or if several domains elicited “some concerns” in a manner that could potentially undermine the validity of the findings.

### Assessing the quality of evidence

The certainty of the evidence concerning the primary outcomes (namely, efficacy, acceptability, and safety) within the network estimates was scrutinized through the application of the Grading of Recommendations Assessment, Development, and Evaluation (GRADE) system (19). Within the purview of the GRADE framework, the quality of evidence is stratified into four tiers: high, moderate, low, and very low. This is premised upon a comprehensive evaluation of factors such as study limitations, inherent risk of bias, inconsistency, indirectness, imprecision, and the potential for publication bias.

### Data synthesis and statistical analysis

This study's principal analysis was conducted using the netmeta and gemtc packages in R 4.3.1. The Standard Mean Difference (SMD) was employed as the effect measure. Statistical significance was denoted by a *P*-value less than 0.05 and a 95% credible interval (CI) that excludes zero. The analytical process was divided into seven sections:

First, a network geometry plot was utilized to assess the strength of the existing evidence, where the size of the nodes was proportional to the number of studies included for each intervention, and the width of the lines connecting the nodes was proportional to the number of trials directly comparing two interventions (20).

Second, we evaluated consistency by comparing whether the intervention effects estimated by direct comparisons were in alignment with those estimated by indirect comparisons.

Third, a standard pairwise meta-analysis was performed for each direct comparison using the DerSimonian-Laird random-effects method (21). The standardized mean difference score was calculated using Cohen's d as the effect size statistic. Effect sizes were interpreted as follows: values ≤0.2 were considered low, 0.2–0.5 moderate, 0.5–0.8 strong, and ≥0.8 very strong. Statistical heterogeneity was assessed with the I^2^ statistic, with interpretation as follows: I^2^ = 0% to 40% signifying unimportant, I^2^ = 30%–60% moderate, I^2^ = 50%–90% substantial, and I^2^ = 75%–100% considerable heterogeneity. The corresponding *p*-values were also considered (17). To understand the size and clinical relevance of heterogeneity, we computed the *τ*^2^ statistic, with interpretation as follows: *τ*^2^ ≤0.14 low, 0.14–0.40 moderate, and ≥0.40 high degree of heterogeneity. These findings were depicted in a league table format.

Fourth, we assessed transitivity by testing whether the syntheses of direct comparisons of interventions were carried out in samples with similar baseline clinical characteristics.

Fifth, the relative ranks of the exercise interventions were estimated using the treatment rankings and graphically represented using ranking plots. Furthermore, the Surface Under the Cumulative Ranking curve (SUCRA) for each intervention was calculated. SUCRA assigns a value between 0 and 1 to simplify the ranking of each intervention in the ranking plots. The best intervention receives a SUCRA value near 1, while the worst intervention receives a value close to 0 (20, 22). These data were also represented using a rank-heat plot according to the SUCRA values (23).

Additionally, to evaluate the robustness of the estimates and ascertain whether a particular study accounted for a large portion of the heterogeneity, a sensitivity analysis was carried out, sequentially omitting individual studies. A separate sensitivity analysis also excluded studies with a high risk of bias.

Finally, to investigate the presence of bias due to small-study effects, a network funnel plot was utilized for a visual inspection of symmetry. This portion was analyzed using Stata 17.0 (Stata, College Station, TX, USA).

## Results

In total, 12 studies (10, 24–34), encompassing 1,075 patients, were incorporated into this investigation, which included one three-arm trial. As delineated in Supplementary Table S1, six of these studies explored CR, another three investigated aerobic exercise, two were focused on Yoga, and two examined HIIT. Lastly, the physical component was assessed in 11 studies, and the mental component was examined in 10 studies.

### Risk of bias

**Figure 2:**
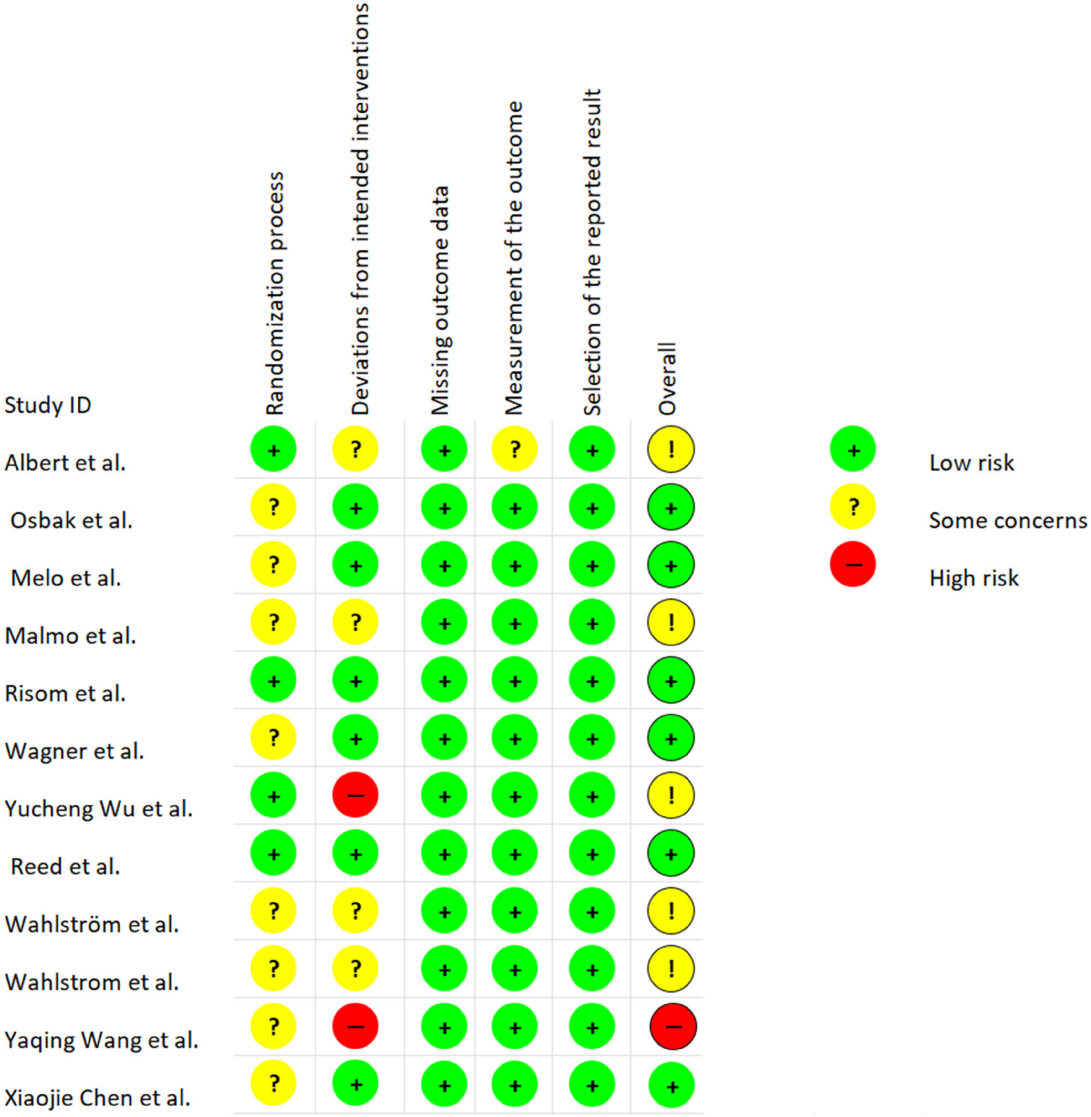
Risk of bias for studies of exercise interventions.

Upon the evaluation through the RoB 2 tool, as depicted in Figure 2, six studies were identified as having low risk, while five studies exhibited some concerns. A single study was classified as high risk. In distinct domains, eight studies demonstrated some concerns regarding the randomization process. Regarding the deviations from intended interventions, four studies displayed some concerns, and two studies were identified with a high risk of bias. Pertaining to the measurement of outcomes, one study expressed certain reservations. The GRADE evaluations are in Supplementary Table S1.

### Network analyze

**Figure 3:**
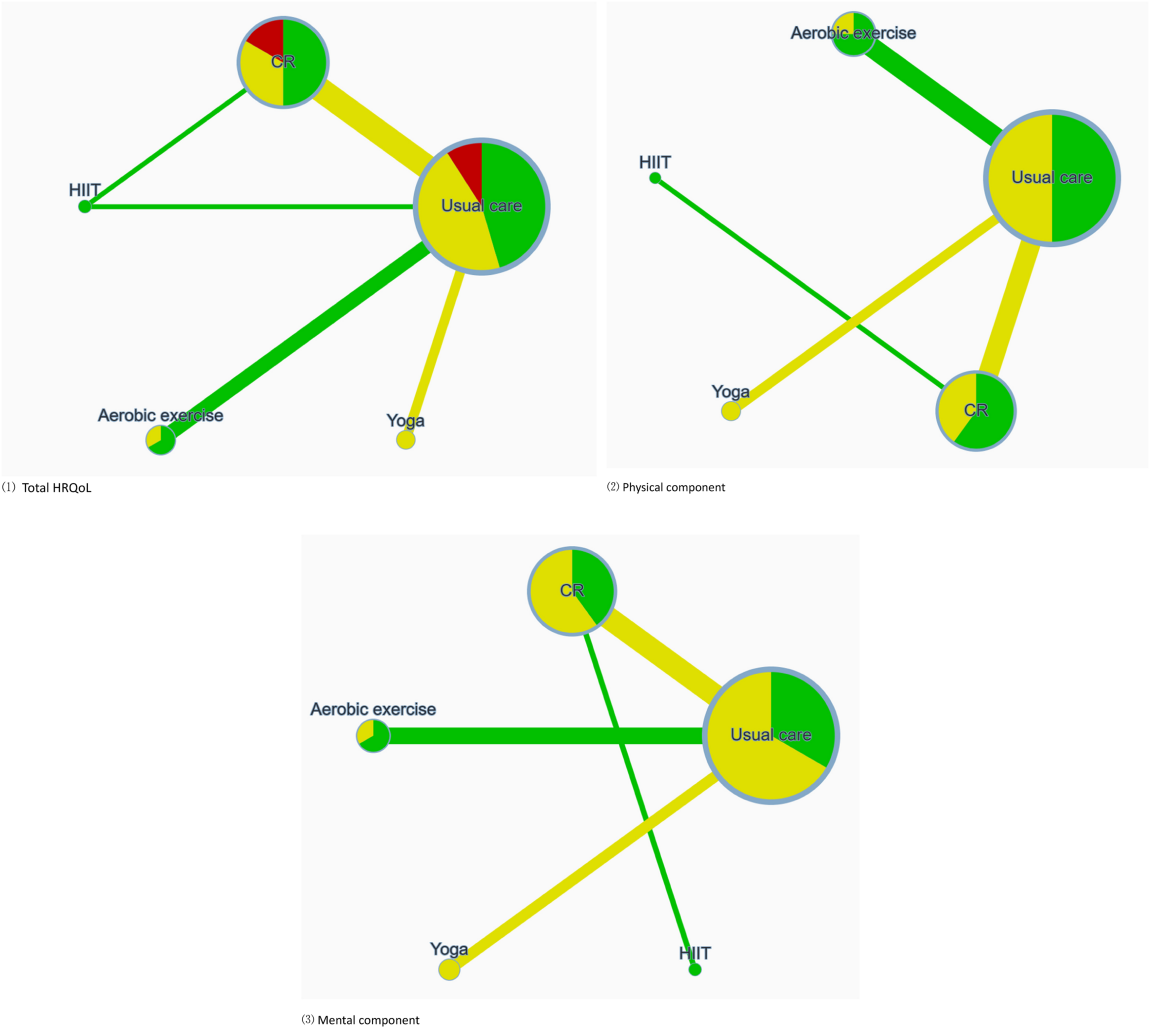
Illustrates the network of available comparisons between different types of exercise interventions for AF, considering three dimensions: (1) total HRQoL; (2) physical component; (3) mental component. Node size reflects the number of trials included for each intervention, with line width indicative of the quantity of studies making a direct comparison between two specific interventions. Risk of bias assessment is depicted through color-coding: green signifies low risk, yellow represents some concerns, and red indicates a high risk of bias. The coloration of connecting lines represents the average risk of bias assessment for the studies making direct comparisons between the two interventions.

The network geometry diagram visualizes the relative volume of available evidence regarding the impact of sports exercise interventions on overall, physical, and mental HRQoL, encompassing 5, 4, and 4 pairwise comparisons respectively. It is essential to note that all interventions have at least one direct comparison with the control group. As shown in Figure 3, the size of the node represents the number of trials included per intervention, with the line width corresponding to the number of studies making direct comparisons between two particular interventions. The risk of bias assessment is depicted via color coding: green signifies low risk, yellow represents moderate risk, and red indicates a high risk of bias. The coloration of the connecting lines signifies the average risk of bias assessment for the studies making direct comparisons between two interventions.

### Modalities of exercise and their effect on physical, mental and total HRQoL

In Table 2, in pairwise comparisons using SMD as the effect measure, no types of exercise interventions demonstrated a significant impact on HRQoL and its related dimensions. However, in the NMA, we found that aerobic exercise and CR had a significant effect on HRQoL, as well as on its physical and mental components. Specifically, aerobic exercise showed the most considerable effect when compared to usual care, with SMD values for HRQoL at 0.60 (95% CI: 0.02, 1.13), for the physical component at 0.47 (95% CI: 0.04, 0.87), and for the mental component at 0.56 (95% CI: 0.00, 1.05).

### Probabilities

**Figure 4:**
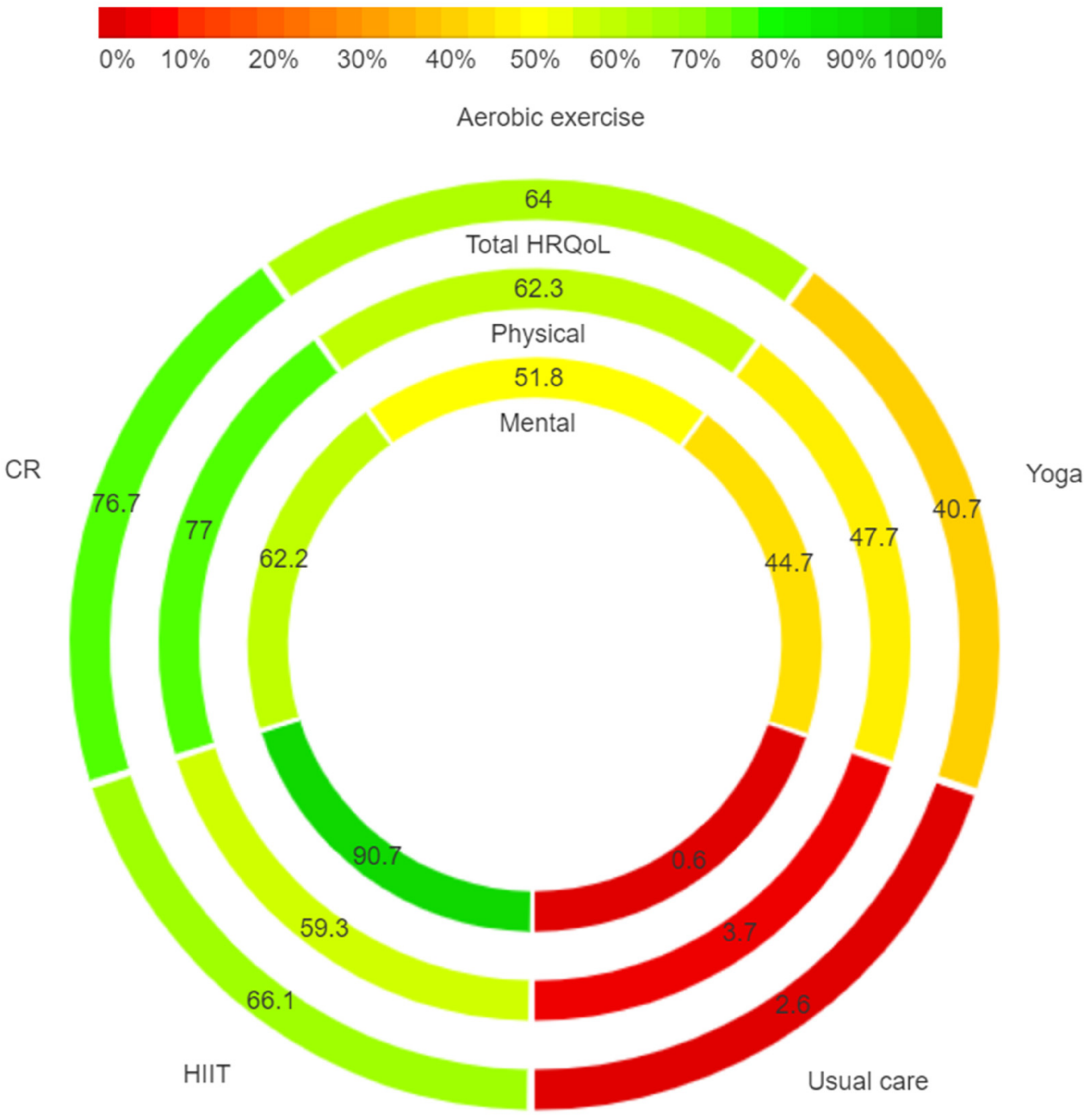
Rank-heat plot with SUCRA values for scoring in total, physical and mental HRQoL. SUCRA, surface under the cumulative ranking curve.

In Figure 4, regarding total HRQoL, the highest SUCRA is observed for CR (76.7%). In the physical component, CR (77.0%) again presents the highest SUCRA. For the mental component, HIIT (90.7%) achieves the highest SUCRA. The ranking diagrams for these are depicted in Supplementary Figure S1.

### Subgroup, sensitivity analysis, heterogeneity, transitivity, and publication bias

Due to the absence of studies for each subgroup, it's impossible to conduct *a priori* subgroup analysis based on the duration, frequency, volume, and intensity of the physical activity interventions.

In the sensitivity analysis, we excluded studies with high risk. Although the pooled SMD experienced some variation, there was no statistically significant alteration in comparison to the NMA incorporating all studies.

The heterogeneity assessment, as shown in Supplementary Table S1, reveals moderate heterogeneity in the total Health-Related Quality of Life (HRQoL) between the Aerobic Exercise and Usual Care groups (I^2^ = 51.1, *τ*^2^ = 0.049). This moderate level of heterogeneity suggests variability in how individuals with atrial fibrillation respond to Aerobic Exercise, potentially influenced by differences in baseline health status, exercise tolerance, or lifestyle factors. In contrast, the comparison between Cardiac Rehabilitation (CR) and Usual Care shows substantial heterogeneity (I^2^ = 75.5, *τ*^2^ = 0.116), implying that the effectiveness of CR varies significantly among different patient populations. This high level of heterogeneity could be attributed to variations in CR program content, intensity, or adherence, as well as differences in patient demographics or comorbid conditions. Regarding the physical component of HRQoL, substantial heterogeneity is also noted between the CR and Usual Care groups (I^2^ = 75.6, *τ*^2^ = 0.113). This indicates that physical health outcomes from CR may vary widely, likely due to differences in patients' physical capabilities, comorbidity burden, or specific exercise regimens within CR programs. For the mental component, moderate heterogeneity is observed in the Aerobic Exercise vs. Usual Care comparison (I^2^ = 43.5), while substantial heterogeneity is present in the CR vs. Usual Care comparison (I^2^ = 62.6). The substantial heterogeneity in CR's effect on mental health suggests that psychological outcomes may vary based on factors such as baseline mental health, support systems, or program engagement levels.

The transitivity assessment, Table 1 provides a comprehensive summary of the included studies, showing comparable characteristics across study populations and interventions. All studies focus on adult populations with similar baseline characteristics, ensuring that age-related variability is minimized. The exercise intervention durations range from 3 to 6 months, with each session lasting no more than one hour. These consistent parameters across studies suggest that populations and intervention durations are sufficiently similar, supporting the assumption of transitivity in this network meta-analysis.

**Table 1:** Characteristics of included studies on the impact of exercise on HRQoL in patients with AF.

Authors, Year	Country	Study design	Age	Exercise duration	Experimental				Comparator				Health-related quality of life scale
					Intervention (N)	Each intervention's duration	Weekly intervention frequency	Training intensity	Intervention (N)	Each intervention's duration	Weekly intervention frequency	Training intensity	
Albert et al., 2019	Denmark	RCT	≥18	6 months	CR (28)	1h	2	Borg: 14–16	Usual care (30)	None	None	None	AF-QoL-18; AFEQT; 5D (EQ-5D)
Osbak et al., 2011	Denmark	RCT	70.2 ± 7.8	12 weeks	Aerobic exercise training (24)	1 h	3	Borg: 14–16	Without training (23)	None	None	None	SF-36; MLHF-Q
Melo et al., 2019	Portugal	RCT	67.6 ± 1.9	6 months	HIIT (7)	1 h	2	Exhaustion, leg fatigue or dyspnea, and RER values >1.1	No specific advice on exercise training and no supervised training (9)	None	None	None	HeartQoL
Malmo et al., 2016	Norway	RCT	59 ± 9	12 weeks	Aerobic Interval Training (26)	1 h	3	HRpeak: 60–95%; Borg: 5–20	Regular exercise habit (26)	None	None	None	SF-36; SSCL
Risom et al., 2016	Denmark	RCT	≥18	6 months	CR (105)	1 h	3	Borg15	Usual care (105)	None	None	None	SF-36
Wagner et al., 2017	Denmark	RCT	≥18	4 months	CR (83)	1 h	3	Borg:13–17	Usual care (90)	None	None	None	SF-36; AFEQT
Yucheng Wu et al., 2022	China	RCT	≥65	6 months	CR (30)	1 h	2–3	Palpitations or shortness of breath	Usual care (33)	None	None	None	SF-36
Reed et al., 2022	Canada	RCT	≥40	3 months	HIIT (43)	23 min	2	Peak power output: 80%–100%	CR (43)	1h	2	HRpeak: 67%–95%;ratings of perceived exertion: 12–16	SF-36
Wahlström et al., 2020	Sweden	RCT (three arms)	64 ± 13	3 months	Yoga (38)	1 h	1	None	relaxation group(29);Usual care (41)	None	None	None	SF-36
Wahlström et al., 2016	Sweden	RCT	64 ± 7	3 months	Yoga (33)	1 h	1	None	Usual care (36)	None	None	None	5D (EQ-5D); SF-36
Yaqing Wang et al., 2019	China	RCT	67 ± 5	18 weeks	CR (50)	30 min	7	Increased heart rate (10–20) beats/min	Usual care (50)	None	None	None	MLWHFQ
Xiaojie Chen et al., 2019	China	RCT	35–70	6 months	Aerobic exercise training (60)	40 min	2–3	HR<160 beats/min; Brog ≤4	Usual care (62)	None	None	None	SF-36

SSCL, the AF symptoms and severity checklist; SF-36,short form 36 health survey; AF-QoL-18: health-related quality of life in patients with atrial fibrillation; AFEQT, atrial fibrillation effect on quality of life; EQ-5D, EuroQol 5D; MLHF-Q, Minnesota living with heart failure; MLWHFQ: Minnesota heart failure quality of life scale; HRpeak, maximal heart rate.

**Table 2:** Absolute and relative effect size estimates for (1) total HRQoL and (2) physical compenent and (3) mental compenent.

Total HRQoL				
Usual care	0.66 (−1.97, 0.68)	0.73 (−1.66, 0.13)	0.00 (−1.7, 1.76)	0.6 (−1.16, 2.32)
**0.60 (0.02, 1.13)**	Aerobic exercise	NA	NA	NA
**0.59 (0.20, 0.99)**	−0.01 (−0.66, 0.71)	CR	−0.08 (−0.88, 0.62)	NA
0.52 (−0.29, 1.27)	−0.08 (−1.06, 0.87)	−0.08 (−0.88, 0.62)	HIIT	NA
0.38 (−0.28, 1.04)	−0.23 (−1.05, 0.68)	−0.22 (−1.00, 0.56)	−0.14 (−1.14, 0.92)	Yoga
Physical compenent				
Usual care	0.32 (1.07, −1.36)	0.52 (−0.12, 1.27)	NA	0.45 (0.78, −1.62)
**0.47 (0.04, 0.87)**	Aerobic exercise	NA	NA	NA
**0.45 (0.08, 0.90)**	−0.02 (−0.55, 0.61)	CR	0.15 (1.11, −1.63)	NA
0.34 (−0.52, 1.30)	−0.13 (−1.06, 0.92)	−0.12 (−0.91, 0.71)	HIIT	NA
0.37 (−0.23, 0.96)	−0.11 (−0.80, 0.63)	−0.08 (−0.84, 0.59)	0.04 (−1.08, 1.06)	Yoga
Mental compenent				
Usual care	0.34 (−1.61, 2.15)	0.73 (−0.19,1.83)	NA	0.66 (−1.02, 2.43)
**0.56 (0.00, 1.05)**	Aerobic exercise	NA	NA	NA
**0.55 (0.14, 1.03)**	−0.01 (−0.63, 0.75)	CR	51.07 (−22.96, 147.8)	NA
0.93 (−0.02, 1.95)	0.37 (−0.71, 1.54)	0.38 (−0.54, 1.25)	HIIT	NA
0.49 (−0.13, 1.12)	−0.07 (−0.86, 0.79)	−0.06 (−0.87, 0.66)	−0.44 (−1.64, 0.70)	Yoga

Upper right triangle gives the effect size from pairwise comparisons (column intervention relative to row); lower left triangle gives the effect size from the network meta-analysis (row intervention relative to column).

Data are effect sizes (95% confidence intervals); bold represents significant statistical significance.

NA, not available; HRQoL: health-related quality of life.

Effect size in bold: statistically significant.

Positive effect sizes mean that the first intervention of the comparison improves quality of life compared to the second intervention.

Finally, the publication bias was identified through the utilization of Egger's test, while comparing the effects of Yoga vs. Usual Care on the mental component (*P* = 0.039). The graphical representation of the bias can be observed in the funnel plots (Supplementary Figure S3).

## Discussion

Despite an abundance of research highlighting the effectiveness of exercise in augmenting the HRQoL in patients with AF, the optimal exercise modality remains elusive. This NMA, which encompasses 12 randomized controlled trials and involves 1,073 participants, offers compelling evidence supporting the role of exercise in improving the HRQoL of AF patients. Through a rigorous review of the current evidence, CR is identified as the most efficacious exercise intervention for enhancing HRQoL in AF patients. CR is identified as the most efficacious exercise intervention for enhancing HRQoL in AF patients. To validate the consistency between direct and indirect comparisons within our network meta-analysis, we implemented several statistical tests. Bucher's test was used to assess local inconsistency within triangular loops in the network, comparing direct effects against the corresponding indirect effects. Additionally, a global consistency test based on the design-by-treatment interaction model was conducted to evaluate overall consistency across the network. These tests indicated no significant inconsistency, reinforcing the robustness of our findings and supporting the reliability of the comparative rankings of exercise interventions in our analysis. Furthermore, both aerobic exercise and CR demonstrate significant improvements in the overall HRQoL, along with distinct physical and mental components, for patients with AF.

As delineated in Figure 4, CR emerges as the preeminent modality of exercise for patients with AF, manifesting superiority across physical and total HRQoL metrics. A wealth of research corroborates the role of exercise-based CR as a vital supplementary intervention in a host of cardiovascular conditions, encompassing myocardial infarction, coronary artery bypass grafting, and heart failure (35–40). Notably, studies from Chinese scholars Yucheng Wu (29) and Yaqing Wang (33) have elucidated the beneficial impact of CR in enhancing cardiorespiratory function, exercise tolerance, and overall quality of life among patients diagnosed with atrial fibrillation. Yet, this consensus is nuanced by a study from Danish researcher Risom (27), who posits that although CR substantially improves physical fitness, it neither confers psychological benefits nor mitigates the risk of adverse events. Complementing this, Albert M's (24) findings reveal that the clinical advantages of CR are relatively transient, with their significance attenuating beyond a 12-month follow-up period.

Aerobic exercise plays a pivotal role in ameliorating human health, particularly in augmenting cardiovascular functionality. Evidence suggests that aerobic activities can mitigate the risk of cardiovascular diseases, including hypertension, hyperlipidemia, and diabetes mellitus, by enhancing endothelial function and attenuating the magnitude of systemic inflammatory responses (41). Nevertheless, the mechanistic implications of aerobic exercise on AF remain a subject of ongoing debate. Osbak (25) et al. observed that aerobic exercise markedly increased exercise capacity and the 6-minute walk test results, while significantly reducing the resting pulse rate in patients diagnosed with AF. Similarly, Malmo (10) et al. reported that a 12-week aerobic exercise regimen reduced AF duration in patients with non-permanent AF, concomitant with substantial improvements in AF symptoms, peak oxygen uptake, left atrial and ventricular function, lipid profiles, and QoL. Conversely, some researchers opine that the exertion from such activities can amplify oxidative stress and inflammatory response intensities, thus facilitating myocardial fibrosis and cardiac tissue remodeling (42). This could potentially elevate the risk of AF onset or exacerbate pre-existing pathological conditions.

In our analysis, HIIT does not exhibit a statistically significant advantage over CR in enhancing physical and total HRQoL for AF patients. However, HIIT demonstrates a notable strength in improving psychological health within this population. Our network meta-analysis shows that while CR achieved the highest SUCRA value for the physical component (77%), HIIT ranked highest for the mental component (90.7%). While SUCRA provides a useful ranking, interpreting these results in a clinical context is essential. CR's high SUCRA ranking for physical health aligns with its proven effectiveness in enhancing cardiorespiratory function, making it ideal for AF management when physical improvement is prioritized. Conversely, HIIT's high ranking for mental health benefits suggests unique psychological effects likely driven by mechanisms such as neuroplasticity, stress reduction, and autonomic regulation. Studies indicate that HIIT increases brain-derived neurotrophic factor (BDNF), which promotes neuroplasticity and resilience, and reduces stress by lowering cortisol levels (43, 44). Furthermore, HIIT's enhancement of cardiovascular autonomic function, through increased parasympathetic activity, may alleviate anxiety and depressive symptoms, supporting its use as a complementary mental health intervention for AF patients (11, 45). These findings suggest that while CR is well-suited for physical health goals in AF patients, HIIT may offer added benefits for mental health. The divergence between our findings and those of Reed et al. (30, 39), who reported lower efficacy of HIIT compared to CR, highlights the need for further research to clarify HIIT's specific psychological impacts in AF populations.

Furthermore, our results offer a counter-narrative to prevailing assumptions regarding the efficacy of yoga in alleviating psychological symptoms among AF patients. Previous studies posited that yoga could mitigate symptoms such as anxiety and depression (46); however, our comprehensive analysis indicates that yoga does not confer any significant advantages over other exercise modalities. In a multi-faceted assessment encompassing physical wellness, psychological well-being, and overall HRQoL, yoga consistently lagged behind the other interventions. As a consequence of these findings, yoga cannot be endorsed as the foremost exercise modality for enhancing the quality of life in AF patients based on the data accumulated in the present study.

Before recommending exercise for AF patients, it is essential to screen for underlying cardiac conditions that could heighten risk during physical activity. Studies highlight that myocardial inflammation and structural abnormalities may exacerbate arrhythmia risk in individuals with pre-existing cardiac issues. Comprehensive diagnostic evaluations, as seen in research on athletes with ventricular arrhythmias, are crucial for identifying high-risk patients and guiding safe exercise prescriptions (47, 48). This approach not only minimizes adverse events but also allows for tailored exercise plans, enhancing both safety and therapeutic efficacy.

Several limitations of our NMA warrant consideration. First, our analysis did not account for the heterogeneity in characteristics of interventions such as intensity, duration, frequency, and timing across the included studies, thereby circumscribing the generalizability of our findings. Second, although we assessed global, physical, and mental domains of HRQoL, we did not explore other potential facets of HRQoL such as pain or sleep, which could act as confounding or mediating variables in the influence of exercise on HRQoL. Third, the inconsistency in the assessment tools utilized across different studies necessitated the use of SMD for estimation, potentially introducing a source of bias in the outcomes. Fourth, our analysis combined patients with different types of AF (paroxysmal, persistent, and permanent) due to limited data, and a subgroup analysis based on AF type was not feasible. This limitation may have influenced our results, as different AF types could respond variably to exercise interventions. Fifth, several studies on overall HRQoL did not disaggregate their findings by specific domains, precluding us from conducting a domain-specific analysis of the impact of exercise interventions on HRQoL. Sixth, the scarcity of available studies, compounded by the small sample sizes in some of the included studies, undermines the reliability of our estimations. Lastly, a substantial proportion of the studies were evaluated to have some concerns (38%) or high risk of bias (7%), primarily due to issues related to the randomization process and deviations from intended interventions. These sources of bias could potentially influence the robustness of the findings by affecting the comparability of intervention groups or introducing unintended effects that may skew results. Consequently, these biases highlight the need for cautious interpretation of the results, as they may impact the reliability and generalizability of the conclusions drawn from this analysis.

In conclusion, exercise emerges as a beneficial modality for enhancing HRQoL in patients with AF. Both CR and aerobic exercise manifest significant efficacy in ameliorating global, physical, and psychological dimensions of quality of life among this patient population. Specifically, CR exhibits superior outcomes in elevating both the overall HRQoL and the physical components, whereas HIIT is most effective in augmenting the psychological domain. Nevertheless, the extant literature in this field is notably limited, warranting further investigation to compare the impact of exercise interventions across varying intensities, frequencies, volumes, and durations. Such additional studies are imperative for a more nuanced understanding of the optimal exercise modalities for improving HRQoL in AF patients.
