# Supplementary material for: The type of exercise most beneficial for quality of life in people with atrial fibrillation: a network meta-analysis
Source: Front Cardiovasc Med. 2025 Jan 9;11:1509304. doi: 10.3389/fcvm.2024.1509304 (PMC11754419; doi:10.3389/fcvm.2024.1509304)
Supplement: Supplementary file 1 [file Image1.pdf]

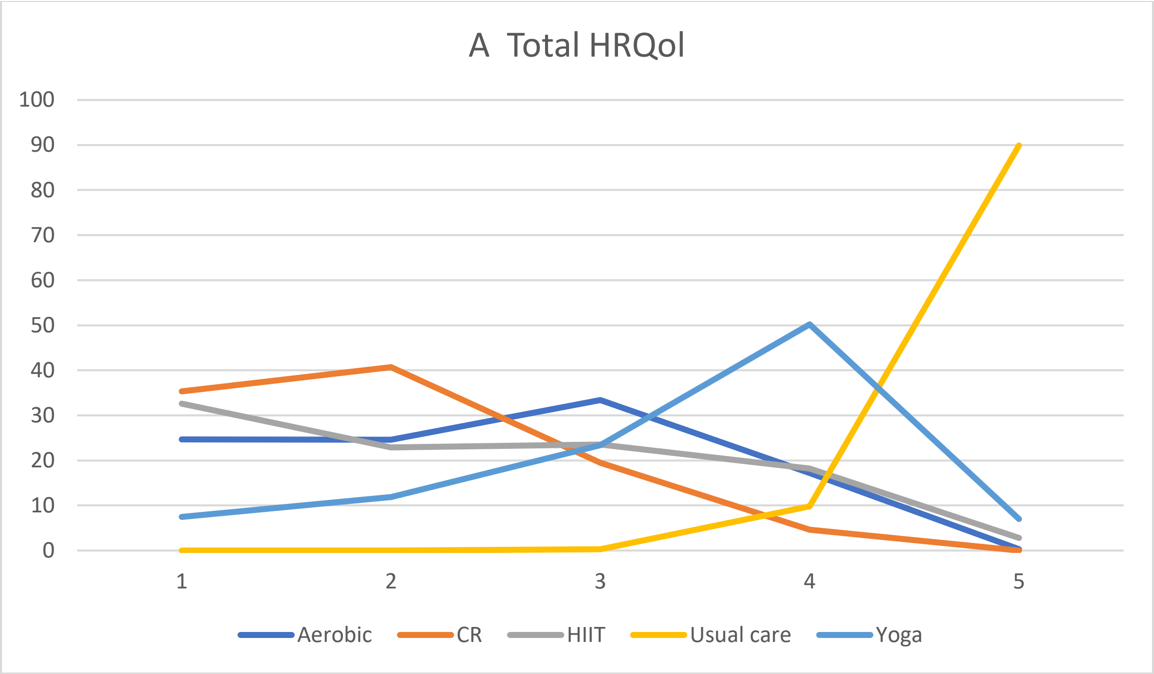
A Total HRQol
100
90
80
70
60
50
40
30
20
10
0
1
2
3
4
5
Aerobic
CR
HIIT
Usual care
Yoga

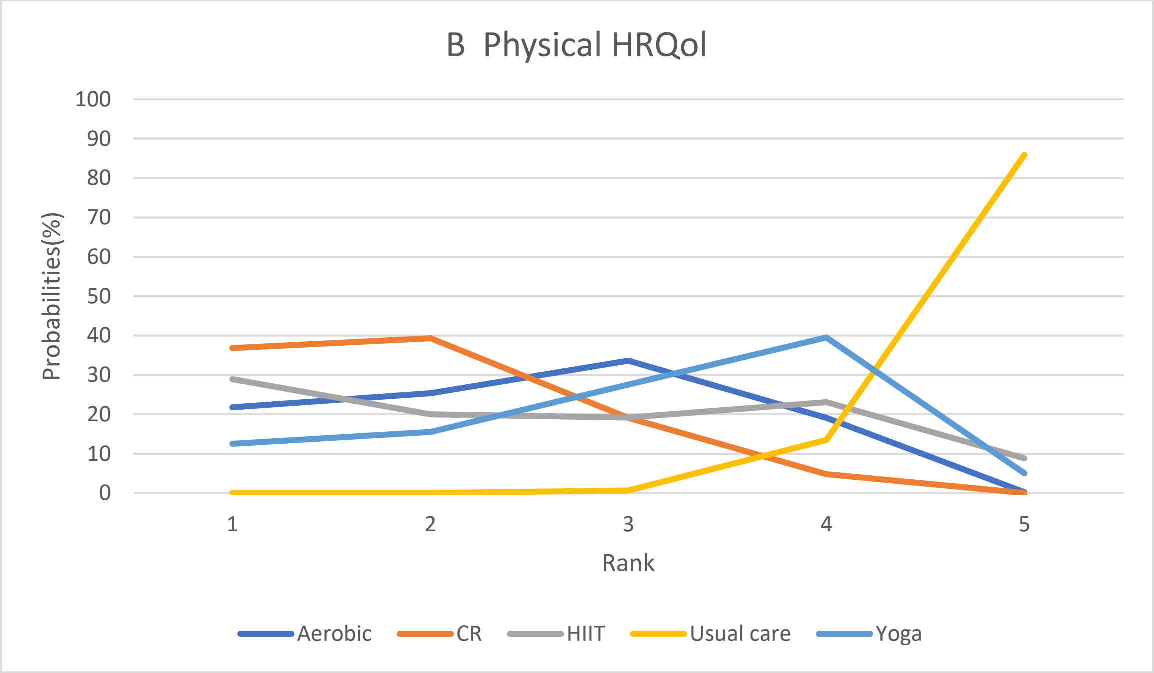
B Physical HRQol
Probabilities(%)
100
90
80
70
60
50
40
30
20
10
0
1
2
3
4
5
Rank
Aerobic
CR
HIIT
Usual care
Yoga

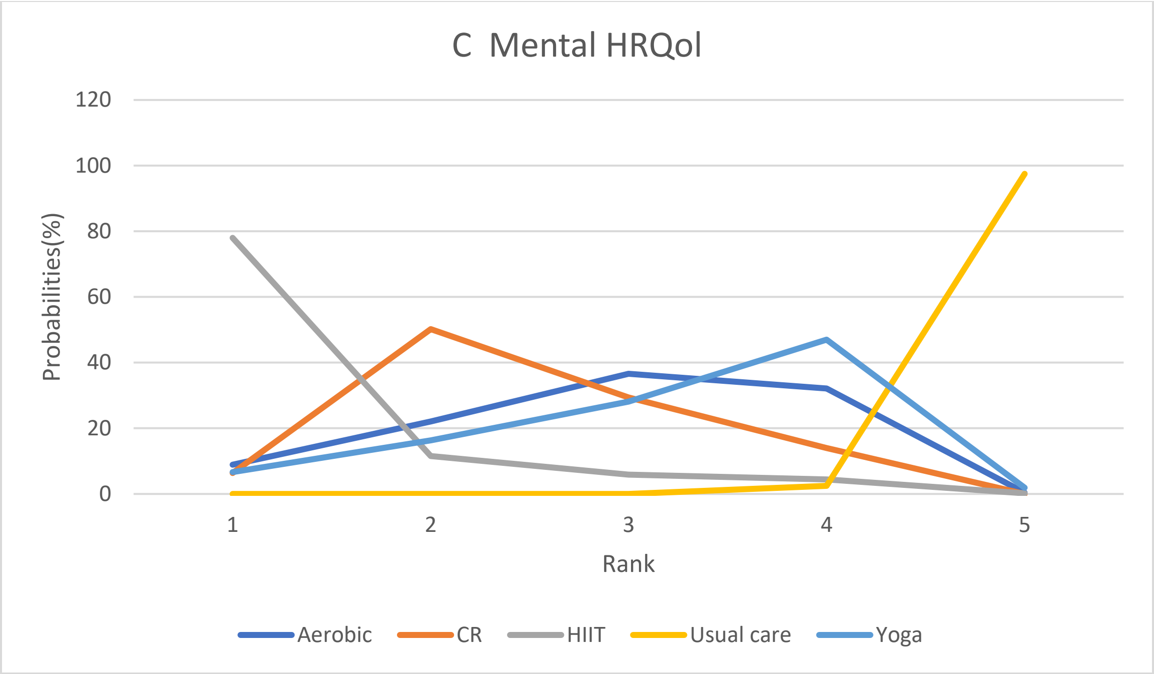

Figure 1S Rankogram for each intervention on HRQoL score in AF patients.

HRQoL, health-related quality of life.
