# Supplementary material for: The type of exercise most beneficial for quality of life in people with atrial fibrillation: a network meta-analysis
Source: Front Cardiovasc Med. 2025 Jan 9;11:1509304. doi: 10.3389/fcvm.2024.1509304 (PMC11754419; doi:10.3389/fcvm.2024.1509304)
Supplement: Supplementary file 2 [file Image2.pdf]

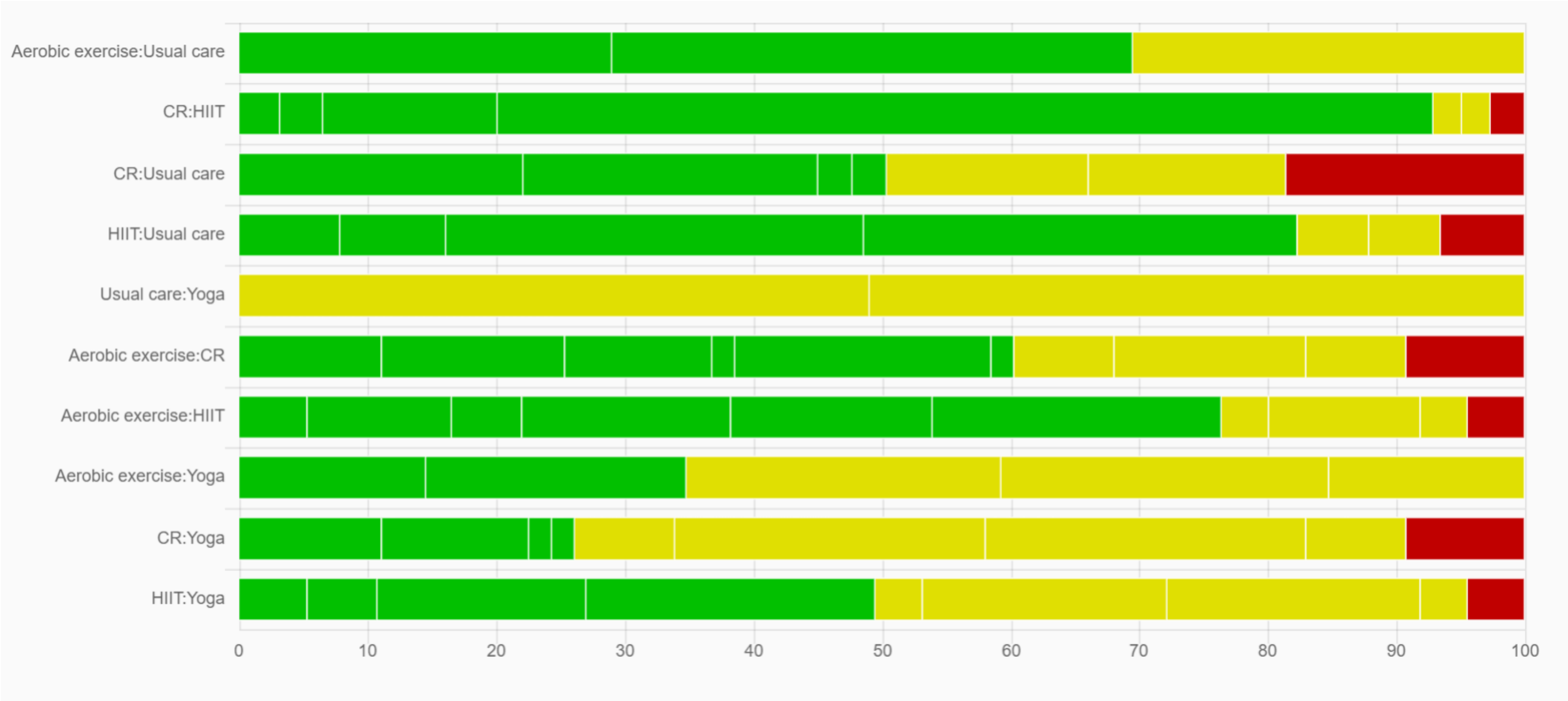

a) Risk of bias contributions for each comparison on total HRQoL . The bar chart shows the contributions of each piece of study to the network estimate.

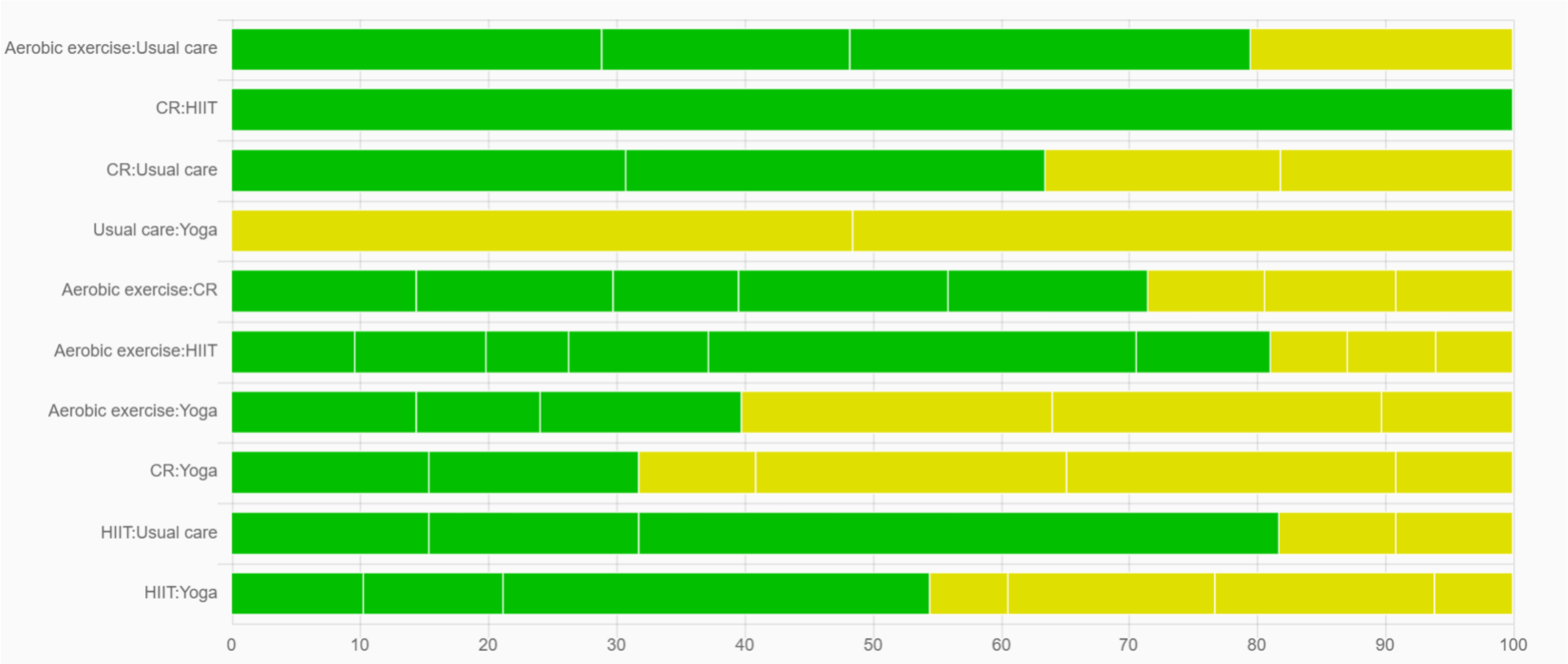

b) Risk of bias contributions for each comparison on physical component . The bar chart shows the contributions of each piece of study to the network estimate.

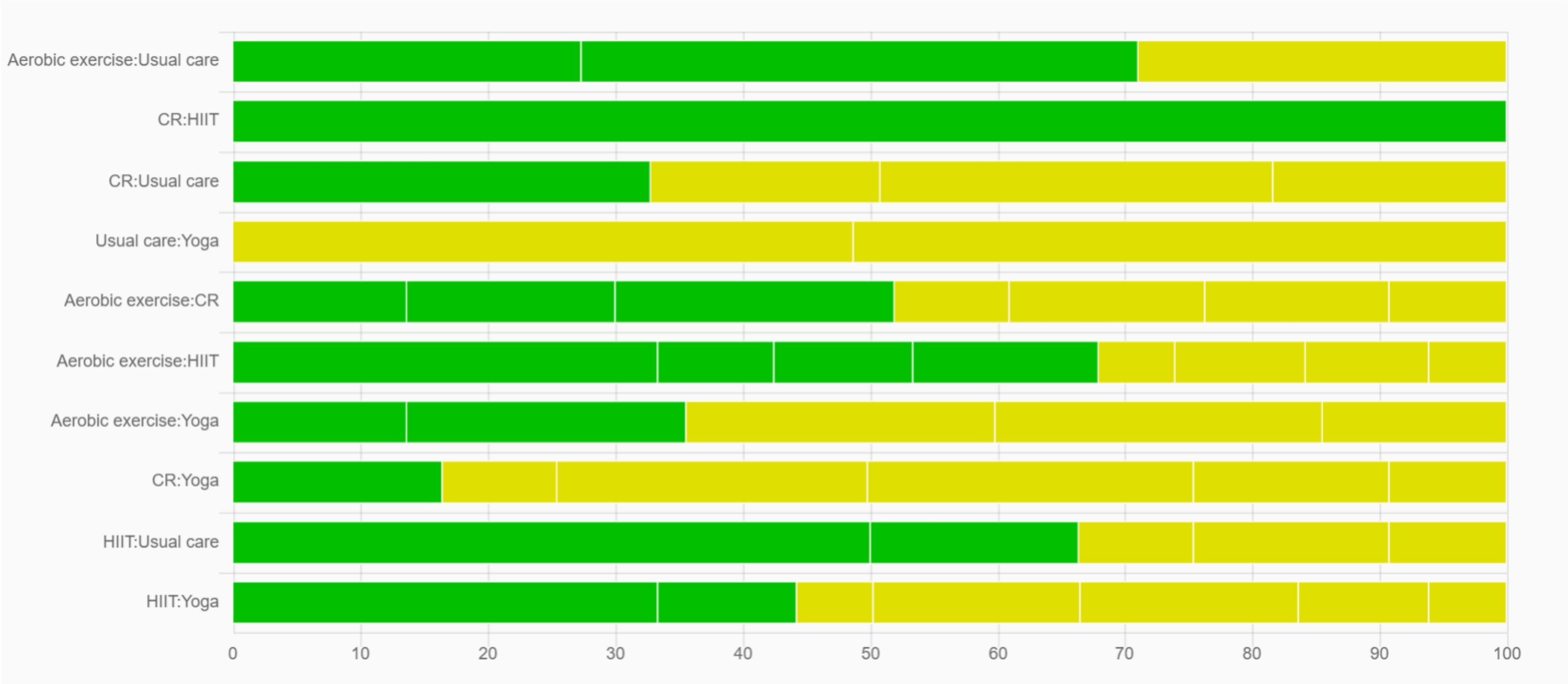

c) Risk of bias contributions for each comparison on mental component . The bar chart shows the contributions of each piece of study to the network estimate.

Figure 2S Risk of bias contributions for each comparison
