# Supplementary material for: The type of exercise most beneficial for quality of life in people with atrial fibrillation: a network meta-analysis
Source: Front Cardiovasc Med. 2025 Jan 9;11:1509304. doi: 10.3389/fcvm.2024.1509304 (PMC11754419; doi:10.3389/fcvm.2024.1509304)
Supplement: Supplementary file 3 [file Image3.pdf]

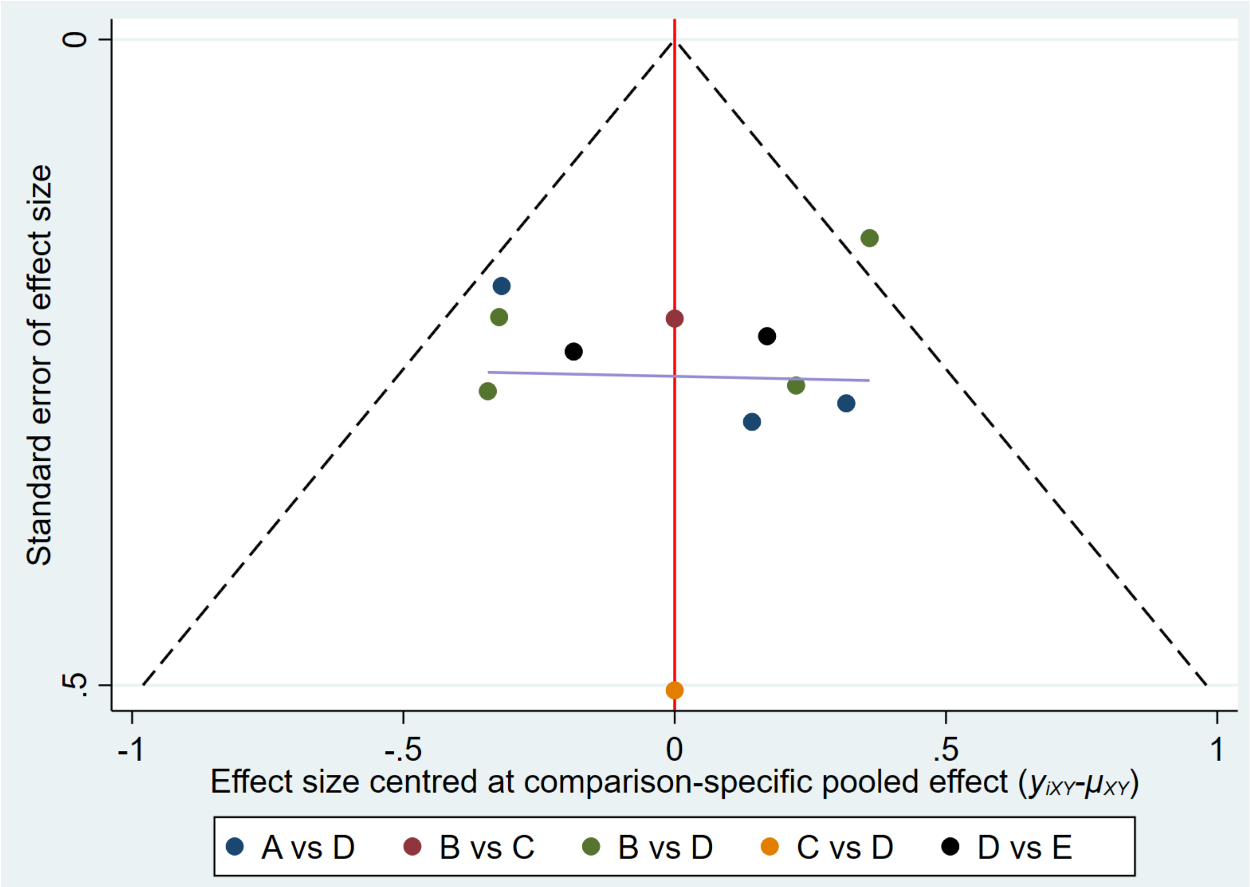

a) Funnel plot for comparison-specific pooled standard mean differences for total HRQoL of AF.

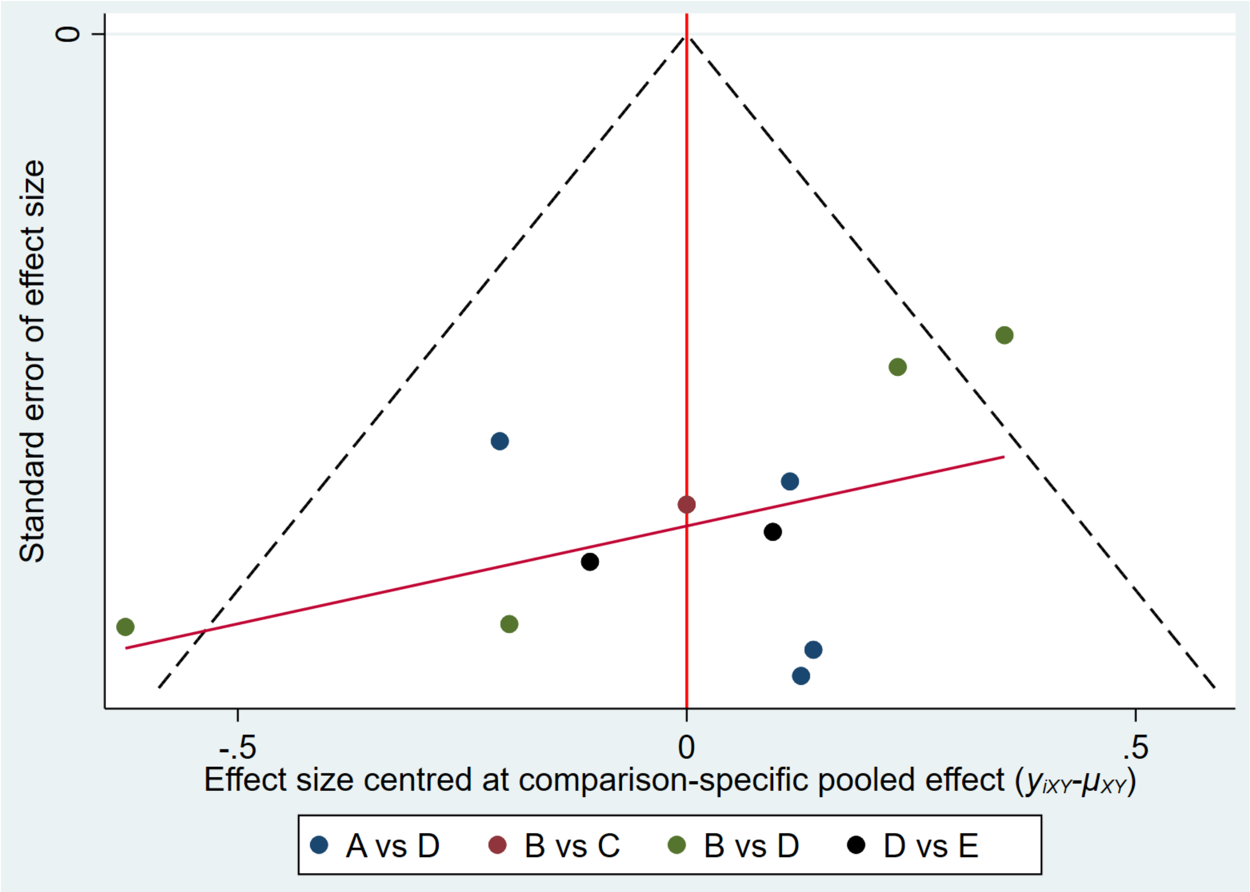

b) Funnel plot for comparison-specific pooled standard mean differences for physical component of AF.

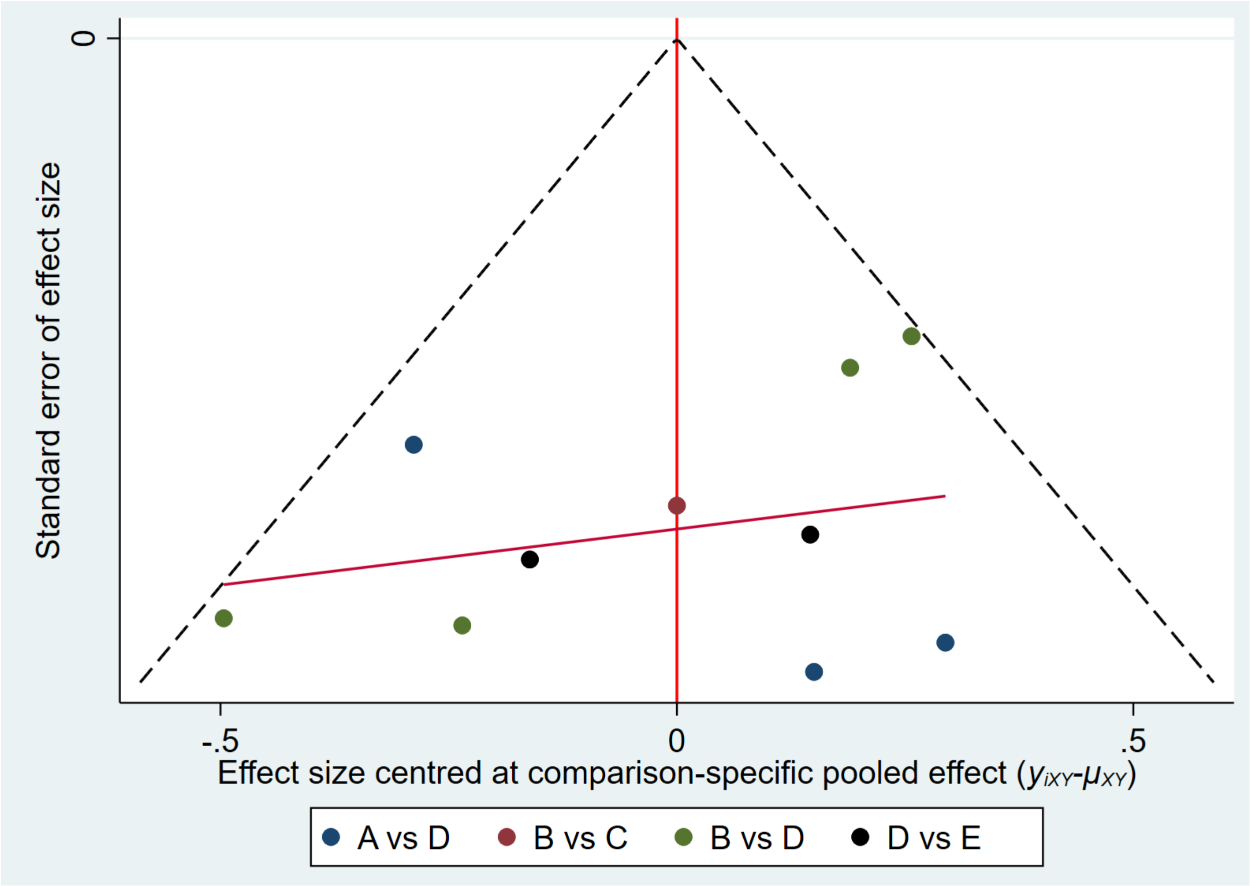

b) Funnel plot for comparison-specific pooled standard mean differences for mental component of AF.

A: Aerobic exercise; B: CR; C: HIIT; D: Usual care; E: Yoga.

Figure 3S   Funnel plot
